# C9orf72-Related Neurodegenerative Diseases: From Clinical Diagnosis to Therapeutic Strategies

**DOI:** 10.3389/fnagi.2022.907122

**Published:** 2022-06-10

**Authors:** Stefania Zampatti, Cristina Peconi, Rosa Campopiano, Stefano Gambardella, Carlo Caltagirone, Emiliano Giardina

**Affiliations:** ^1^Genomic Medicine Laboratory UILDM, IRCCS Fondazione Santa Lucia, Rome, Italy; ^2^IRCCS Neuromed, Pozzilli, Italy; ^3^Department of Biomolecular Sciences, University of Urbino “Carlo Bo”, Urbino, Italy; ^4^Department of Clinical and Behavioral Neurology, IRCCS Fondazione Santa Lucia, Rome, Italy; ^5^Department of Biomedicine and Prevention, Tor Vergata University of Rome, Rome, Italy

**Keywords:** neurodegeneration, C9orf72, ALS, FTD, therapeutic strategies, molecular mechanisms

## Abstract

Hexanucleotide expansion in *C9orf72* has been related to several phenotypes to date, complicating the clinical recognition of these neurodegenerative disorders. An early diagnosis can improve the management of patients, promoting early administration of therapeutic supportive strategies. Here, we report known clinical presentations of *C9orf72*-related neurodegenerative disorders, pointing out suggestive phenotypes that can benefit the genetic characterization of patients. Considering the high variability of *C9orf72*-related disorder, frequent and rare manifestations are described, with detailed clinical, instrumental evaluation, and supportive therapeutical approaches. Furthermore, to improve the understanding of molecular pathways of the disease and potential therapeutical targets, a detailed description of the cellular mechanisms related to the pathological effect of *C9orf72* is reported. New promising therapeutical strategies and ongoing studies are reported highlighting their molecular role in cellular pathological pathways of *C9orf72*. These therapeutic approaches are particularly promising because they seem to stop the disease before neuronal damage. The knowledge of clinical and molecular features of *C9orf72*-related neurodegenerative disorders improves the therapeutical application of known strategies and will lay the basis for the development of new potential therapies.

## Introduction

Firstly discovered in 2011, the hexanucleotide G_4_C_2_ expansion in the noncoding region between exons 1a and 1b of *C9orf72* gene was identified as the mutation related to the complex phenotype associated with the 9p21 locus (Vance et al., 2006). Characteristically, pedigrees in which the mutated allele of *C9orf72* segregated from one generation to another, showed different affected phenotypes, ranging from amyotrophic lateral sclerosis (ALS) to frontotemporal dementia (FTD) sometimes with peculiar features (DeJesus-Hernandez et al., 2011; Renton et al., 2011). In detail, patients carrying the high-repeat expansion (HRE) in *C9orf72* showed a pure ALS phenotype, a pure FTD phenotype (mostly behavior variant), or a combination of both. Interestingly, about 35% of patients with HRE *C9orf72* receive a diagnosis at the onset of the disease that is different from ALS or FTD. This happens because about one in three patients shows an atypical presentation at onset, these different presentations often mime other neurodegenerative disorders (i.e., Alzheimer’s, Parkinson’s, and Huntington’s disease, dementia w/Lewy bodies, vascular dementia, atypical Parkinsonian syndromes, neuropsychiatric disorders, etc; Gossye et al., 2020; Moore et al., 2020). Clinically, there are rapid and slow progressive forms and the phenotype at the onset of the disease is sometimes misleading (van der Ende et al., 2021). The overall survival ranges between 2 months in rapidly progressive ALS to over 30 years in slowly progressive behavior variant of FTD (bvFTD).

The frequency of HRE *C9orf72* is extremely variable worldwide, with a great prevalence in the European population, while it is almost absent in the Asian population. Frontotemporal dementia has a prevalence of 1–461:100,000. Among all FTD patients, a percentage of about 4%–29% carry an HRE *C9orf72* (Hogan et al., 2016; Van Mossevelde et al., 2018). In detail, about 20%–25% of familial-FTDs and 6%–8% of sporadic-FTDs carries an HRE *C9orf72* (van Es et al., 2017; van der Ende et al., 2021). Amyotrophic lateral sclerosis has a prevalence of 5–12:100,000. In detail, one in 10 cases of ALS has a positive family history, and about 30%–50% of ALS familial cases carry the HRE *C9orf72* allele. Even in sporadic ALS, the HRE *C9orf72* allele is greatly represented in (about 4%–10% of all sporadic ALS). Over 30% of patients manifesting both ALS and FTD phenotypes carried an HRE *C9orf72* allele (van der Ende et al., 2021).

These frequencies support the phenotypic variability and incomplete penetrance of some HRE *C9orf72* alleles (Oskarsson et al., 2018; Masrori and Van Damme, 2020). In particular, the HRE *C9orf72* exhibits incomplete and age-dependent penetrance. A large study conducted by Murphy and coworkers reported that the median age at symptom onset is 58.0 years of age, with a younger age at onset for ALS than for FTD. Indeed, the youngest affected patient described was a 23-year-old patient with ALS. The penetrance increased with age and became nearly complete (99.5%) over 83 years of age (Murphy et al., 2017). Interestingly, the familial and sporadic cases showed the same penetrance of HRE *C9orf72*. In this scenario, it is hypothesized that almost all sporadic cases are unrecognized familial cases. This assumption is based on the increased life expectancy over the past decades. In fact, the average life expectancy in the United States was under 58 years until the 1930s. In the early decades of the 1900s, people with a HRE *C9orf72* allele had the same probability of dying from a common disease rather than from a *C9orf72* associated phenotype. This phenomenon is called Gompertzian inter-disease competition and explains the increased frequency of ALS in the past century, due to the effective increase in the susceptible population (Riggs, 1990). *C9orf72* phenotypes showed great variability in age at the onset, even in the same family. This fact is perhaps due to the different possible phenotypes at onset. In fact, it is not surprising that motor disturbance (prevalent in ALS forms) is easily recognized if compared with language and/or behavior changes (prevalent in FTD forms).

## C9orf72 Phenotypes

Characteristically defined as ALS and FTD phenotypes, the clinical presentation of *C9orf72* associated disorders may vary from pure ALS to pure FTD, with many intermediate phenotypes. In fact, the motor neuron disease–FTD continuum encompasses three ALS forms: ALS with executive dysfunction (ALS-eci), ALS with cognitive impairment (ALS-neci), and ALS with behavioral modifications (ALS-bi). Furthermore, FTD presentations may vary from the most frequent behavioral variant (bvFTD) to the primary progressive aphasias (PPAs), which are in turn subdivided in a non-fluent variant (nfvPPA), semantic variant (svPPA), and logopenic variant (van Es et al., 2017).

### C9orf72-FTD Phenotype

Frontotemporal dementia represents a group of syndromes characterized by progressive impairment of behavior, language, and cognitive functions. The FTD is due to a frontotemporal lobar degeneration (FTLD) and is clinically subdivided into behavioral variant (bvFTD), semantic variant of primary progressive aphasia (svPPA), and non-fluent/agrammatical variant of primary progressive aphasias (nfvPPA). Among all FTD cases with an HRE *C9orf72*, there is a higher prevalence of bvFTD cases. The average age at onset is 57 years, regardless of the *C9orf72* genotype. Progression is faster in C9-FTD cases if compared with non-C9-FTDs, with overall survival of 7 years in C9-FTD vs. 11 years in non-C9-FTDs, regardless of the occurrence of a concomitant ALS (van Es et al., 2017; van der Ende et al., 2021). Furthermore, up to 30% of C9-FTD patients develop motoneuron dysfunction, a substantially overlapping proportion to FTD cases regardless of C9 genotypes (Burrell et al., 2011).

Cognitive features associated with FTD involve early loss of executive functions, mnemonic deficits, and language impairment. Other clinical features may become evident with the disease progression like dyscalculia and apraxia, suggesting the involvement of parietal lobe (Van Mossevelde et al., 2018; Moore et al., 2020).

The clinical presentation of bvFTD includes early behavioral disinhibition, early apathy or inertia, early loss of sympathy or empathy, and early perseverative, stereotyped, or compulsive or ritualistic behavior. Patients often modify their food preferences, generally with a predilection for sweets. Other neuropsychiatric features are early delusions (mostly somatic) and hallucinations, psychosis, and anxiety. The PPA is characterized by early language deficits, with the impairment of the ability to understand oral and written language. Patients affected by svPPA forget word significance, with the inability to name the objects. On the other hand, patients affected by nfvPPA (agrammatical) lose the ability to apply grammar rules in oral and written language. They lose the ability to understand complex phrases and sometimes present pronunciation disturbance (apraxia) (Van Mossevelde et al., 2018; Moore et al., 2020).

At disease onset, about 90% of all C9-FTD patients are diagnosed as bvFTD, 5% as nfvPPA, and 3% as svPPA. The remaining 2% receive a diagnosis of other neurodegenerative disorders (corticobasal degeneration, progressive supranuclear palsy, etc.; Moore et al., 2020).

Although clinical presentation of C9-FTD and non-C9-FTD are very similar and no clinical criteria can support the differential diagnosis between the two groups, some differences have been described. In particular, mnemonic impairment is more frequent in C9-FTD patients and is often the onset symptom. Psychoses and bizarre behaviors are common in C9-FTD patients. Neuroimages show different degrees of atrophy ranging from the generalized symmetric cortical atrophy to the thalamus and cerebellum atrophy. Furthermore, some C9-FTD patients manifest quite a non-progressive benign variant of the disorder. The benign variant of bvFTD is slowly progressive, with an apparent steadiness of the clinical appearance, very few psychiatric symptoms, and cognitive decline. It is estimated that about 2% of patients with a benign variant of bvFTD carry an HRE *C9orf72* allele (van der Ende et al., 2021).

The occurrence of mnemonic impairment at the onset of C9-FTD disease can confuse the diagnosis and lead to a misdiagnosis of Alzheimer’s disease (AD). It is important to consider that AD is a frequent disorder, and it is possible that C9 patients manifest FTD with concomitant AD. Furthermore, C9 patients may not manifest FTD in reason of an incomplete penetrance, independently from the occurrence of other dementing disorders. The small percentage (<1%) of HRE *C9orf72* alleles described in AD patients is probably due to the phenotypic heterogeneity of C9-FTD (that involves mnemonic dysfunction) leading to misdiagnosis, and to the known incomplete penetrance (Cacace et al., 2013; Harms et al., 2013). In this scenario, it is important to consider *C9orf72* expansion testing in dementing patients with a positive family history of ALS and/or FTD (Harms et al., 2013).

### C9orf72-ALS Phenotype

Amyotrophic lateral sclerosis (ALS) is a neurodegenerative progressive disorder that affects the brain and the spinal cord. Although ALS is traditionally considered a motoneuron disease, it can also involve frontal and temporal lobes. Furthermore, the clinical overlap has been demonstrated with other diseases (Paget’s disease of bone, inclusion body myopathy, etc.). Typically, the clinical picture involves a motor impairment with some symptoms of cognitive decline and/or behavior changes. ALS is a disorder with a high phenotypic heterogeneity regarding disease presentation, progression, and survival.

The prevalence of ALS is 5–12:100,000, with a slight reduction in European populations (2.6–3:100,000). Besides phenotype classifications (bulbar, spinal, pseudobulbar palsy, progressive spinal muscular atrophy), ALS can be divided into sporadic and familial forms according to the age at onset and the family history. The median age at onset of ALS is quite similar between sporadic and familial cases: about 65 years for sporadic ALS, and about 55 years for genetic-related familial ALS (Beck et al., 2013; Bieniek et al., 2014; Fahey et al., 2014; Block et al., 2016; Glasmacher et al., 2020). The hexanucleotide expansion in *C9orf72* has been recognized as one of the most frequent causes of familial ALS, but it is frequent also in sporadic patients. The classical clinical onset of ALS is the occurrence of muscle weakness in a segment that successively progresses to the whole motor system. Clinically, the classic ALS (70% of cases) has a presentation at the onset that involves both upper motor neuron and lower motor neuron signs. The distribution of the disease is bulbar (33% of classic ALS) with dysarthria and dysphagia at onset, or spinal (66% of classic ALS) with lower and upper motor neuron involvement in the arms and legs. About 5%–15% of ALS patients manifest at onset cognitive or behavioral changes, and it is classified as ALS-FTD. Other rare phenotypes (10% of cases) present a generalized lower motor neuron involvement at onset (progressive spinal muscular atrophy) or an exclusive upper motor neuron involvement (primary lateral sclerosis) (van Es et al., 2017). As seen in the C9-FTD, the C9-ALS is clinically similar to non-C9-ALS, with a few differences in the frequency of some symptoms and signs between the two forms. In particular, the bulbar onset (dysarthria and dysphagia) is more frequent in C9-ALS patients than in non-C9-ALS, with about 30%–40% of all C9-ALS patients presenting bulbar signs at the onset. Neuroimages of C9-ALS patients show a major atrophy of non-motor frontal cortex areas if compared with non-C9-ALS. Cognitive decline is more common in C9-ALS patients than non-C9-ALS. Despite only 20% of ALS patients fulfill diagnostic criteria for FTD, another 20% of ALS patients show cognitive decline (generally in executive, social, or linguistic functions) and a further 10% present behavior changes. This clinical overlap confirmed the phenotype continuum between FTD and ALS.

### Atypical Presentations

Psychiatric symptoms are very frequent in patients with a *C9orf72*-associated disorder if compared with non-C9-FTD and non-C9-ALS. In particular, 20%–60% of the patients carrying the HRE *C9orf72* allele manifest psychotic signs, above all delusions (mostly somatic), and hallucinations (Benussi et al., 2021). Other psychiatric manifestations are behavior disturbance, obsessive-compulsive disorder, and catatonia. Sometimes psychiatric symptoms are the first manifestation of the *C9orf72* disorder, preceding the clinical diagnosis even for many years. Attention must be paid to the atypical late onset of a psychiatric disorder (without prodromal signs at typical ages) because it can be misdiagnosed as late schizophrenia or bipolar disorder instead of *C9orf72* disease. In fact, the identification of an HRE *C9orf72* allele is very rare in primary psychiatric disorders (about 0.2%). On the other hand, the identification of psychotic symptoms in C9-FTD patients is very frequent, covering about 21%–56% of bvFTD and FTD-ALS patients (Silverman et al., 2019; van der Ende et al., 2021). In this scenario, the *C9orf72* hexanucleotide expansion should be genotyped in all suspected late onset (over 40 years of age) psychiatric disorders in which the symptoms are accompanied by cognitive decline, cerebral or cerebellar atrophy, or familiar history of FTD, ALS, or psychiatric disorders.

Another atypical onset of *C9orf72* disease is a Parkinsonian disorder. Although, parkinsonism is a common clinical sign of *C9orf72*-associated diseases, described in over 75% affected people, sometimes Parkinsonism is a unique sign at onset, or it is only accompanied by ataxia or apraxia. Some HRE *C9orf72* carrying people show at onset bradykinesia, rigidity, postural instability, gaze paralysis, generally without tremors. Typical FTD or ALS signs became manifest several years after the onset of parkinsonism, leading to a misdiagnosis of Parkinson’s disease, progressive supranuclear palsy, corticobasal syndrome, or multisystemic atrophy (Wilke et al., 2016; van der Ende et al., 2021). Movement disorders are very frequent in patients with an HRE *C9Orf72* allele: tremors and parkinsonism are described in over 60% of patients, myoclonus, dystonia, and chorea in 30%–40% of patients, and ataxia in less than 10% (Estevez-Fraga et al., 2021). Even in this scenario, a *C9orf72*hexanucleotide expansion genotyping should be considered in patients showing atypical parkinsonism and: (i) family history of ALS or FTD, or; (ii) motoneuron sign, and cognitive impairment with frontotemporal involvement.

Another interesting onset phenotype is the Huntington’s disease-like (HDL) syndrome. In particular, about 5% of patients with HDL syndrome (clinically indistinguishable from HD, but without the CAG expansion in *HTT*) carries an HRE *C9orf72* allele. Although, a study has reported CAG expansions in FTD/ALS patients without HRE *C9orf72* allele, further studies have to be performed in order to explain this peculiar overlap between *C9orf72* and *HTT*-associated phenotypes (van der Ende et al., 2021).

## Pathological Mechanisms

**Figure 1:**
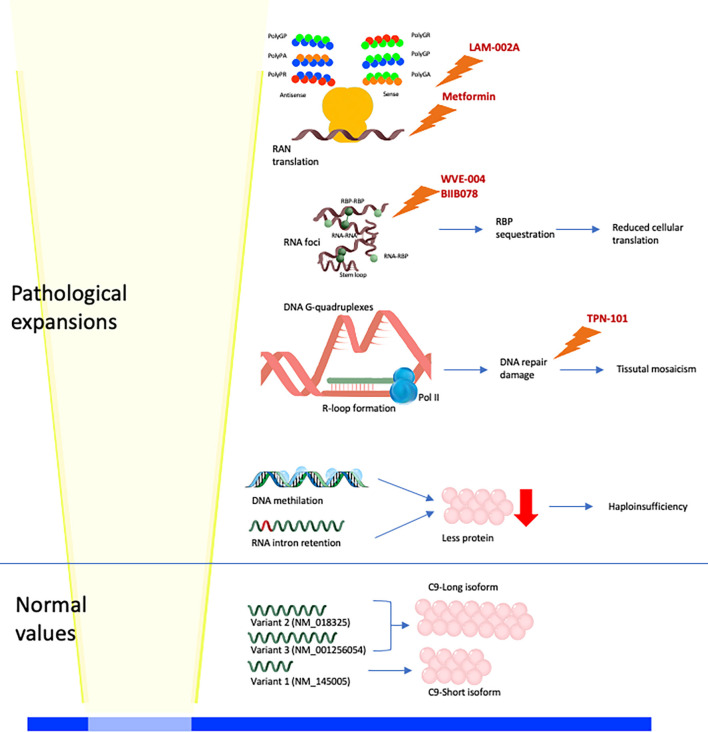
Physiological and pathological molecular mechanisms linked to C9orf72 expansion. Normal alleles lead to the production of three mRNA (V1, V2, and V3), translated into two C9 isoforms (long and short; Smeyers et al., 2021). High-repeat expansion (HRE) *C9orf72* alleles lead to four different pathological mechanisms: (i) hypermethylation of DNA and intron retention in mRNA leading to haploinsufficiency; (ii) DNA G-quadruplexes and R-loop formation leading to activation of DNA repair damage response and somaticmosaicism; (iii) RNA foci formation, with intramolecular (stem-loop, and G-quadruplexes) and intermolecular (RNA-RNA, RNA-RNA-binding protein (RBP), and RBP-RBP) interactions (Malik et al., 2021), leading to RBP sequestration and reduced cellular translation; (iv) RAN translation of sense and antisense mRNAs produces five different DPRs (polyGA, polyGR, polyGP, polyPR, and polyPA). Drugs of which it is known or supposed molecular function are reported: metformin reduces RAN translation, LAM-002A interferes with DPRs formation, WVE-004 and BIIB078 promotes the degradation of C9orf72 expanded mRNAs, TPN-101 reduces the DNA repair damage response.

Three different etiopathological mechanisms have been proposed as responsible for the cellular impairment leading to *C9orf72* disorder: haploinsufficiency (low expression of the HRE *C9orf72* allele), RNA-binding protein (RBP) sequestration, and the toxic function of RNA-foci and DPRs (dipeptide repeats; Figure 1). These three mechanisms synergistically interplay. The hexanucleotide expansion of *C9orf72* determines intron retention and haploinsufficiency of wild-type C9orf72. Furthermore, the HRE *C9orf72* allele shows a hypermethylation of the promoter region with a consequent transcriptional silencing of the expanded allele and partial to complete absence of translation of the expanded allele (haploinsufficiency). The transcription of the HRE *C9orf72* allele leads to the formation of RNA-DNA hybrids (R-loops). R-loops activate DNA damage response, which interferes with the expansion and is potentially responsible for the somatic variability of the *C9orf72* expansion. Moreover, the long repeat RNA has a longer half-life than wild-type RNA, determining the sequestration of the RNA-binding proteins (RBPs; Malik et al., 2021). Sense and antisense long repeat RNAs and RBP interact to form RNA foci. RNA foci sequestrate RNA-binding proteins compromising the overall translation cellular activity. Moreover, intramolecular, and intermolecular interactions recognized in RNA foci (as G-quadruplexes) can further activate DNA damage response. Another mechanism of the disease involves the repeat associated non-AUG translation (RAN translation) of sense and antisense mRNAs from C9orf72 hexanucleotide (Figure 1). The RAN translation produces five different dipeptide repeat proteins (DPRs): polyGA (glycine–alanine), polyGR (glycine–arginine), polyGP (glycine–proline), polyPR (proline–arginine), and polyPA (proline–alanine). The DPRs exercise variable toxic function on tissues. In particular, polyRP shows the highest level of toxicity on several cellular substrates, on the other hand, polyPA shows the lowest level of toxicity (Semmelink et al., 2021).

As described, the phenotypes associated with an HRE *C9orf72* allele are variable. To date, one of the hypotheses at the basis of this great phenotypic variability is the somatic variability of hexanucleotide expansion. As seen, DNA damage response and maybe other molecular mechanisms determine the contraction or the expansion of the hexanucleotide alleles. In fact, a recent study evaluated the levels of expansion in *C9orf72* on brain and blood tissues, finding no correlation between the blood and brain (frontal cortex and cerebellum) exists. In the same person, cells with small and large expansion size can coexist in different tissues (somatic mosaicism). A *C9orf72*-associated phenotype is typically observed in people with a large expansion in cerebral tissue. However, the sizing of the hexanucleotide repeat in the blood may not match the sizing in the brain. On the contrary, people with a large expansion in blood may have small expansion sizes in the cerebral tissues, resulting in milder C*9orf72* phenotypes (Jackson et al., 2020).

These tissue differences support the molecular diagnostic algorithm that defines only normal and pathological ranges, without a clear definition of intermediate alleles. It is hypothesized that the complexity to define a genotype-phenotype correlation among intermediate alleles is mainly due to the extreme variability of the size of the expansion in different cerebral and extra-cerebral tissues (van Blitterswijk et al., 2013 ; Van Mossevelde et al., 2017; Jackson et al., 2020).

## Molecular Algorithms

To date, several molecular algorithms have been proposed to make a diagnosis in neurodegenerative disorders related to *C9orf72*. One of the most diffused involves the deep clinical evaluation of the patient and their family history, in order to administer a first-tier test that involves *C9orf72* hexanucleotide evaluation. Patients without HRE *C9orf72* alleles will be then evaluated for a multigene test (second tier) or whole-exome sequencing (third tier; Roggenbuck, 2020). This effective algorithm (Roggenbuck and Fong, 2020) must consider the different distribution of pathogenic mutations worldwide (Moore et al., 2020). In fact, although, in almost all Caucasian and Hispanic populations *C9orf72* is an important cause of the disease, it is quite inexistent in Asian populations. In this scenario, the first evaluation of a patient with a clinical presentation that overlaps with typical or atypical *C9orf72* phenotypes should consider not only the family history for related disorders but also the ethnical background, in order to prioritize the genetic test (*C9orf72* hexanucleotide evaluation, multigene panel, or whole-exome sequencing).

## Therapeutic Strategies

The management of *C9orf72*-related disorders requires a complete clinical and instrumental evaluation of the patient in order to identify appropriate treatment strategies. In detail, after the diagnosis, the patient should undergo: (i) neurologic examination (upper or lower motor neuron involvement, dysarthria, dysphagia), (ii) neuropsychological evaluation (cognitive assessment), (iii) ADL (activities of daily living) assessment (orthopedics, physiatrist), (iv) psychiatric evaluation (if history of psychiatric illness), (v) pulmonologist evaluation (if respiratory impairment). Therapeutic strategies involve the treatment of the disease manifestations: rehabilitation and riluzol/edaravone for UMN/LMN involvement and ADL, muscle stretching and baclofen/tizanidine/cannabinoids for spasticity, dietary supplements and drugs for muscular cramps, cognitive rehabilitation for cognitive impairment, neuropsychiatric therapies (pharmacological and psychological) for behavioral manifestations, rehabilitative treatment for dysphagia and dysarthria, pharmacological treatments for Parkinsonism, pseudobulbar signs, sialorrhea, bladder dysfunction (Gossye et al., 2020).

To date, 29 research studies on *C9orf72*-related disorders are recorded in ClinTrials.gov. Twelve of them investigate new and off-label drugs.

### Off Label Drugs

#### Metformin

A Single-Center, Open Label Study at the University of Florida (NCT04220021) is studying the safety and potential efficacy of metformin for the treatment of C9-ALS/FTD. Metformin is a widely used drug for type 2 diabetes treatment. At a molecular level, metformin has demonstrated an activity to block the double-stranded RNA-dependent protein kinase (PKR) pathway (Zu et al., 2020). Furthermore, in a mouse model of C9-ALS/FTD metformin reduces neuronal RAN protein levels without modifications to total mRNA C9orf72 levels. A functional analysis of mouse models demonstrated that metformin treatment can improve the behavioral phenotype (Zu et al., 2020).

#### Deferiprone

A study on ALS patients (regardless of *C9orf72* genotype) is evaluating the potential neuroprotective benefits of an iron chelator, deferiprone (NCT03293069). It is expected that deferiprone will reduce excess iron from brain regions, oxidative damage, and cell death.

#### Baricitinib

An open-label study (NCT05189106) is evaluating the potential effects of baricitinib therapy on patients with subjective cognitive disorder, mild cognitive impairment, Alzheimer’s disease (AD), Amyotrophic lateral sclerosis (ALS), or asymptomatic carriers of an ALS-related gene (as hexanucleotide expansion in the *C9orf72*), with evidence of abnormal inflammatory signaling in cerebrospinal fluid (CSF) at baseline. The objective of the study is to evaluate the ability of baricitiniband to reduce neuroinflammatory damage. Baricitinib is approved by the FDA in the United States for rheumatoid arthritis (2 mg per day) and has an emergency use authorization for COVID-19 (4 mg per day).

#### Triumeq

A Double-Blind Placebo-Controlled trial is evaluating the effectiveness and safety of triumeq (dolutegravir 50 mg, abacavir 600 mg, lamivudine 300 mg) in delaying the progression of ALS (NCT05193994). Triumeq therapy is expected to reduce neuroinflammation and human endogenous retroviruses (HERV) impairment, that may have a role in the pathophysiology of ALS. A preliminary study has reported a decline in ALS progression with triumeq therapy (Gold et al., 2019). Triumeq is approved in US and Europe for HIV treatment.

### New Investigational Drugs

#### TPN-101

An ongoing phase 2a study is evaluating the safety and tolerability of TPN-101 on a small sample (40 participants) of *C9orf72* ALS/FTD patients (NCT04993755). TPN-101 (OBP-601, BMS-986001, festinavir, censavudine; Smith et al., 2015) is a thymidine analog NRTI (nucleoside reverse transcriptase inhibitor) that shows major efficacy against HIV-2 and HIV-1 (Gupta et al., 2016). The active form of TPN-101 (BMS-986001-5’-triphosphate) is a substrate for incorporation by HIV-1 reverse transcriptase, inducing the termination of viral DNA synthesis (Smith et al., 2015).

#### LAM-002A

An ongoing phase 2a study on 12 adults with *C9orf72*-associated ALS (C9ALS) is evaluating the safety, tolerability, and biological effect of LAM-002A (NCT05163886). LAM-002A (apilimoddimesylate) reduce glutamate-induced neuronal loss inhibiting PIKFYVE [a lipid kinase that regulates endolysosomal trafficking, via conversion of phosphatidylinositol 3-phosphate PI3P in phosphtidylinositol (3,5)-bisphosphate PI(3,5) P2].

It is hypothesized that the inhibition of PIKFYVE activity may improve the autophagosome-lysosome fusion and increase the PI3P (phosphatidylinositol 3-phosphate) that leads to removal of glutamate receptors and DPRs. A study on human-induced motor neurons (iMNs) revealed that apilimod increases the iMNs survival of *C9orf72* patients (Shi et al., 2018). Similarly, apilimod shows a significant activity in the reduction of the neuronal death in *MAPT* mutated cells (Bowles et al., 2021) and in ATP7A iMNs (Bakkar et al., 2021).

#### AL001

An ongoing clinical trial on patients with *C9orf72*-associated ALS (NCT05053035) is evaluating the safety, tolerability, pharmacokinetics, and pharmacodynamics of AL001. Another study (NCT03987295) is evaluating AL001 in symptomatic carriers of *GRN* mutation or hexanucleotide repeat expansion in *C9orf72* causative of FTD. AL001 is an anti-sortilin antibody that binds sortilin 1 (SORT1), a transmembrane glycoprotein that improves progranulin (PGRN) clearance through cellular internalization. Anti-sortilin antibodies downregulates SORT1 inducing an up-regulation of PGRN (Miyakawa et al., 2020). Previous studies have shown the ability of AL001 to increase progranulin cerebral levels reducing the motor neuron damage TDP-43 associated. Similarly, *GRN*-related FTD AL001 reduces neurodegeneration (Haynes et al., 2020; Terryn et al., 2021).

#### WVE-004

WVE-004 is an antisense oligonucleotide (ASO) that binds the hexanucleotide expansion of *C9orf72* promoting the degradation of *C9orf72* expanded mRNAs. In this scenario, the reduction of mutated mRNA could improve many molecular mechanisms involved in the pathogenesis of *C9orf72* disorders. *In vitro* and *in vivo* studies on patient-derived induced pluripotent stem cells (iPSC) and transgenic mice showed the reduction of expanded transcripts in iPSC motor neurons and in the spinal cord and cortex, respectively (Liu et al., 2022). An ongoing double bind, placebo-controlled, study (NCT04931862) is expected to evaluate the safety, tolerability, pharmacokinetics, and pharmacodynamics of WVE-004 in patients with *C9orf72*-associated ALS or FTD.

#### BIIB078

BIIB078 is an antisense oligonucleotide (ASO) targeting expanded *C9orf72* mRNAs. BIIB078 accelerates the degradation of mutated mRNA, preventing the production of RNA foci and DPRs. Preclinical studies on cellular and murine models showed a reduction of sense RNA foci, and of poly(GP) and poly(GA) proteins. Furthermore, ASOs seems to attenuate behavioral impairment (Lagier-Tourenne et al., 2013; Jiang et al., 2016). An extension study on BIIB078 (NCT04288856) is ongoing on adults with *C9orf72*-associated ALS, previously treated in the double-bind study (NCT03626012).

#### PU-AD

PU-AD belongs to a class of drugs named epichaperome inhibitors. It is a molecular inhibitor of heat shock protein 90 (HSP90), a stabilizer of modified proteins in cancer and neurodegenerative disorders. In cellular and animal models of different neurodegenerative disorders, HSP90 inhibitors promote the degradation of pathogenic proteins (Carman et al., 2013). Epichaperomes are stable HSP90-centered chaperone complexes that incorporate proteins not physiologically associated with HSP90. Among these proteins, there are some involved in synaptic function, learning, and memory. PU-AD has demonstrated to be effective in disrupting the epichaperomes restoring physiological association protein-HSP90 (Inda et al., 2020).

A randomized double-blind placebo-controlled study (NCT04505358) is evaluating the effectiveness and safety of PU-AD therapy on ALS patients. It is a pilot study on 30 participants, randomized 3:2 (PU-AD vs. placebo, respectively).

## Conclusions

From the first recognition of the hexanucleotide expansion in *C9orf72* as the causative mutation of several neurodegenerative phenotypes, numerous studies have been conducted elucidating phenotype heterogeneity, penetrance, pathogenic molecular mechanisms, and treatment strategies. To date, a deep knowledge of the disorder is essential to improve the clinical practice, to provide an early diagnosis and new molecular therapies design. The knowledge of different onset phenotypes and the availability of a genetic test to confirm the diagnosis can improve the management of patients, providing an early administration of therapeutic strategies. Besides lifestyle interventions (Tsitkanou et al., 2019; Hautbergue et al., 2021; Julian et al., 2021), several therapeutic agents are being studied. Interestingly, many new interventional treatments are designed to act at a molecular level preventing neuronal damage even before the onset of symptoms. The potential effectiveness of these drugs in delaying disease onset in *C9orf72* neurodegenerative disorders is designing an optimistic scenario for affected families. The advances in knowledge of clinical and molecular features of *C9orf72*-related neurodegenerative disorders will promote the designing of new molecular therapies, the early treatment administration, and the development of studies on possible off-label effectiveness of medications.

## Author Contributions

SZ and EG designed the theme of the manuscript. SZ, CP, and RC contributed by writing all the sections and creating the figure. EG, SG, and CC conducted critical revisions of the manuscript. All authors contributed to the article and approved the submitted version.

## Conflict of Interest

The authors declare that the research was conducted in the absence of any commercial or financial relationships that could be construed as a potential conflict of interest.

## Publisher’s Note

All claims expressed in this article are solely those of the authors and do not necessarily represent those of their affiliated organizations, or those of the publisher, the editors and the reviewers. Any product that may be evaluated in this article, or claim that may be made by its manufacturer, is not guaranteed or endorsed by the publisher.
